# News from South Africa

**Published:** 1921-01-22

**Authors:** 


### NEWS FROM SOUTH AFRICA.

### St. Helena Hospital Fund.

THE "St. Helena Guardian" has the following respecting the island's Hospital Emergency Fund: "This deserving fund, which has been kept going for twelve years purely by voluntary contributions, is, we understand, in very low water. That it is under the control of the Hon. W. J. J. Arnold, our popular Colonial Surgeon, is a recommendation in itself, and we hope that some of our public-spirited friends will make an effort to float the fund once more on a good, substantial basis."

### Johannesburg General Hospital.

A JOHANNESBURG journal, in a leading article under the heading "Our Great Hospital," says that the work of the Johannesburg Hospital is going up by leaps and bounds. Ten years ago the bed accommodation was 389. At the close of the present financial year it was 740 (including 147 at the three branches). In the same period the daily average of patients has gone up from 371.8 to 614.62, while the paid staff has gone up from 299, including 30 nurses, to 661, including 290 nurses, the corresponding figures for the honorary visiting staff being 16 and 34. Patients dealt with this year alone number 46,000, a daily average of 585 for the last three months, giving a bare idea of the work done. Expenditure has risen from £48,000 in 1909-10 to £137,000, the cost per patient being 7s. 0¼d. and 11s. 9.68d. respectively.

Such figures clearly indicate the importance of proceeding immediately with the necessary extension. The nurses' home is an especial need. £20,000 has already been voted, but a like additional sum is wanted to complete the scheme.

The proposal to establish correlation between the medical faculty of the University College and the Hospital, which is now being considered, will, when adopted, have a wide and beneficial effect on medical knowledge on the Rand.

### Natal Hospitals.

THE Administrator of Natal, Mr. G. T. Plowman, says that, considering its small white population, and the character of the country, Natal can congratulate itself on what has been accomplished in regard to hospitals. The provision of funds by the Provincial Administration for hospitals is on most liberal lines, so much so that probably in no other part of the world is the financial support so favourable as in Natal. Since the date of Union (1910) the cost of hospital maintenance alone in Natal—to say nothing of capital expenditure and recurring charges in respect of interest and sinking fund—has increased from £25,000 to £84,000.

